# Rationale and design of active play @ home: a parent-led physical activity program for children with and without disability

**DOI:** 10.1186/1471-2431-14-41

**Published:** 2014-02-14

**Authors:** Daniela A Rubin, Kathleen S Wilson, Lenny D Wiersma, Jie W Weiss, Debra J Rose

**Affiliations:** 1 Department of Kinesiology, California State University, 800 N. State College Blvd., KHS-138, Fullerton, CA 92834, USA; 2 Department of Health Sciences, California State University, Fullerton, CA USA

**Keywords:** Prader-Willi syndrome, Obesity, Childhood, Family, Interactive games, Playground games, Exercise routine

## Abstract

**Background:**

Compared to other children, those with disability have additional challenges to being physically active. Prader-Willi Syndrome is a genetic form of childhood obesity that is characterized by hypotonia, growth hormone deficiency, behavioral, and cognitive disability. In children, the low prevalence of this syndrome (1 in 10,000 to 15,000 live births) makes group-based physical activity interventions difficult. In contrast, the home environment presents a natural venue to establish a physical activity routine for this population. This manuscript describes the design of a parent-led physical activity intervention incorporating playground and interactive console-based games to increase physical activity participation in youth with and without Prader-Willi Syndrome.

**Methods/Design:**

The study participants will be 115 youth ages 8-15 y (45 with the syndrome and 70 without the syndrome but categorized as obese). The study will use a parallel design with the control group receiving the intervention after serving as control. Participants will be expected to complete a physical activity curriculum 4 days a week for 6 months including playground games 2 days a week and interactive console games 2 days a week. Parents will be trained at baseline and then provided with a curriculum and equipment to guide their implementation of the program. Tips related to scheduling and coping with barriers to daily program implementation will be provided. Throughout, parents will be contacted by phone once a week (weeks 1-4) and then every other week to receive support in between visits. Measurements of children and parents will be obtained at baseline, 12 weeks, and at the end (week 24) of the intervention. Children main outcomes include physical activity (accelerometry), body composition (dual x-ray absorptiometry), motor proficiency (Bruininks-Oseretsky Test of Motor Proficiency), quality of life and physical activity self-efficacy (questionnaires). Intervention compliance will be monitored using mail-in daily self-report checklists.

**Discussion:**

This parent-guided physical activity intervention aims to increase physical activity by using a curriculum that builds physical activity related self-confidence through the development and/or enhancement of motor skill competency. Ultimately, helping children develop these skills as well as joy in being physically active will translate into sustained behavior change.

**Trial registration:**

Current Controlled Trial: NCT02058342

## Background

The U.S. Department of Health and Human Services, the Subcommittee of the President’s Council on Fitness, Sports & Nutrition and the American College of Sports Medicine, among others, recommend that children and youth participate in 60 minutes a day of moderate to vigorous physical activity (PA) [1,2]. It is further recommended that the content of the PA include aerobic, muscle, and bone-strengthening activities [2]. Similar recommendations have been put forward to stimulate the participation of children with disability in sports and recreational activities as well as to achieve the same expected 60 minutes-a-day of moderate-to-vigorous PA (MVPA) [3]. Recommendations reside on the fact that PA provides numerous health benefits for children such as helping regulate blood sugar, insulin, lipids and blood pressure, maintaining a healthy weight, developing stronger bones, building muscle mass and improving physical fitness [2,4,5]. Additionally PA is associated with increased self-efficacy, less depression and anxiety and ultimately a higher quality of life [2].

Currently, 58% of children ages 6-11 y are not meeting these recommended guidelines [6]. And, available studies in children and adolescents with physical and/or cognitive disability suggest that their participation in PA may be lower [7-11] and does not meet the recommended minutes [11]. Low participation in PA has been linked to the increasing prevalence of obesity in youth with disability [7].

Prader-Willi Syndrome (PWS) is a rare congenital disease stemming from an alteration or the lack of expression of the paternal chromosome 15 in the locus 13-15q. This syndrome is the best-characterized form of childhood obesity and people with PWS have abnormally high body fat percentage and low lean mass [12]. In addition, having PWS is associated with innate lethargy, delayed motor development, lower motor competencies, lower cognitive function, and behavioral challenges, with most individuals presenting with physical and intellectual disability [13]. Children and adults with this condition exhibit low levels of ambulatory PA [14,15], little vigorous PA, and appear to perform few activities aimed at improving muscular strength [16]. In children with PWS, more PA has been associated with lower body mass index (BMI), and reduced engagement in self-injury behavior common to PWS such as skin picking [17]. To date, four approaches to promoting PA in persons with PWS have been evaluated: two strength-training routines delivered at home for children [18,19], and a walking and an at home strengthening programs for adults [20,21]. Although these programs were successful at improving body composition, spontaneous physical activity and general fitness levels, none of these approaches considered the multiple dimensions of physical fitness (e.g., aerobic endurance, strength, flexibility), the development and improvement of motor competencies, or the concept of developing a fun family PA routine.

### Theoretical framework

Social Cognitive Theory was the theoretical framework used to guide the development and implementation of this physical activity intervention [22]. Self-efficacy is a focal determinant of Social Cognitive Theory and has often been a key target of PA interventions [23]. For individuals to adopt and maintain a healthy lifestyle including behaviors such as PA, the person needs to have the self-regulatory skills and the confidence to regulate the behavior [24]. Self-regulatory skills (i.e. self-monitoring, planning, coping with barriers) have been related to improving PA behavior by increasing adherence in adults [25] and often are included in interventions that employ a social cognitive theory framework [26].

Individuals may manage or regulate their own behavior and/or have another individual serve as a proxy to manage their behavior [27]. In children, parents may serve as a proxy and aid in the management and regulation of their child’s PA by scheduling opportunities for PA and providing equipment and/or transportation. Further, it might be speculated that in children with disability the parent may take a more active role in regulating the child’s behavior. The primary assumption of Social Cognitive Theory is that behavior, environment, and the person are reciprocally linked [28]. A key part of children’s environment involves the influence from parents as they play an essential role in the development of their children’s behaviors, attitudes, and values. In fact, previous studies have shown that parental influences are associated with children being more physically active [29]. Parents have the potential not only to serve as proxy agents for their children’s PA but also to directly influence their children’s perceptions and behaviors. By targeting aspects of the person (e.g., PA self-efficacy) and the environment (e.g., social influences received from parents), an associated change in the child’s physical activity may emerge.

### Successful approaches to increase PA in children

In children without disability, only a small number of interventions have been designed to improve motor skills [30,31] since children who are more proficient in different motor domains (e.g., agility, balance, coordination, bilateral coordination, muscle strength, and aerobic endurance) are likely to be more physically active [32-34]. Activity cards or manuals containing progressive games and exercises have been successfully used in school settings and after school programs to increase MVPA and improve cardiovascular fitness [35,36]. Most recently, interactive console-based games have been used to increase PA [37,38] and even promote weight loss [39]. In those with disability, the use of interactive console-based games seems a promising area of study as positive results in gross motor function have been shown for programs in children with cerebral palsy [40,41] as well as Down syndrome [42].

Studies targeting lifestyle changes in children with obesity, inclusion of the family has been identified as a key component in effective interventions [43]. In fact, in children with disability, family participation in PA has been shown to be a positive predictor of the child’s PA [9]. Unfortunately, only a few studies have explored the feasibility of family interventions [44-46]; some interventions have targeted solely the parents as agents of change [47-49] and other interventions have included parent-child dyads participating in home-based PA programs [50,51]. Co-activity, in which parents and children participate in physical activity together, is positively associated with PA in children [52], and thus appears to be important to target in a PA intervention. A recent report on strategies to promote PA in youth highlighted the need to test strategies that can take place at home and involve the parents or family [2].

### The development of the active play @ home curriculum

Considering the abilities, needs, constraints, and preferences of children with PWS and their parents as well as previous intervention approaches that proved successful in this population [18,19] we developed a home-based PA curriculum called Active Play @ Home. This well-rounded and varied PA curriculum includes all the essential exercise components recommended in the national guidelines (progressive games and exercises targeting cardiovascular fitness, muscular strength and endurance) while also targeting motor skill competencies. In addition, the curriculum incorporates the use of interactive console-based games that were carefully chosen to stimulate fitness components as well as specific motor skill competencies. The curriculum therefore blends more traditional playground games and exercises with interactive console-based games. The inclusion of the interactive console-based games aims to provide the children with a choice of activities that can be performed indoors while the playground games can be played outdoors. The curriculum is designed to involve an adult leader and one child; however, all activities can be played with more than one child in the home environment.

The goal of the Active Play @ Home study is to determine if a parent-led PA curriculum incorporating playground games and interactive console-based games can increase levels of PA and lead to positive motor and health-related outcomes in children with PWS and in children without the syndrome but who are obese. Changes in PA and motor and health-related parameters will be evaluated in these two groups in comparison to control groups following a 24-week PA intervention. The primary hypothesis is that an age-appropriate home-based PA intervention will increase PA levels in children with and without PWS. The secondary hypotheses include the following: 1) motor proficiency, central sensory reception and integration, and body composition will significantly improve in children, with and without PWS, following completion of the home-based PA intervention, and 2) self-efficacy and quality of life will increase significantly in children, with and without PWS, who complete the home-based PA intervention.

## Methods/Design

### Study design

**Figure 1:**
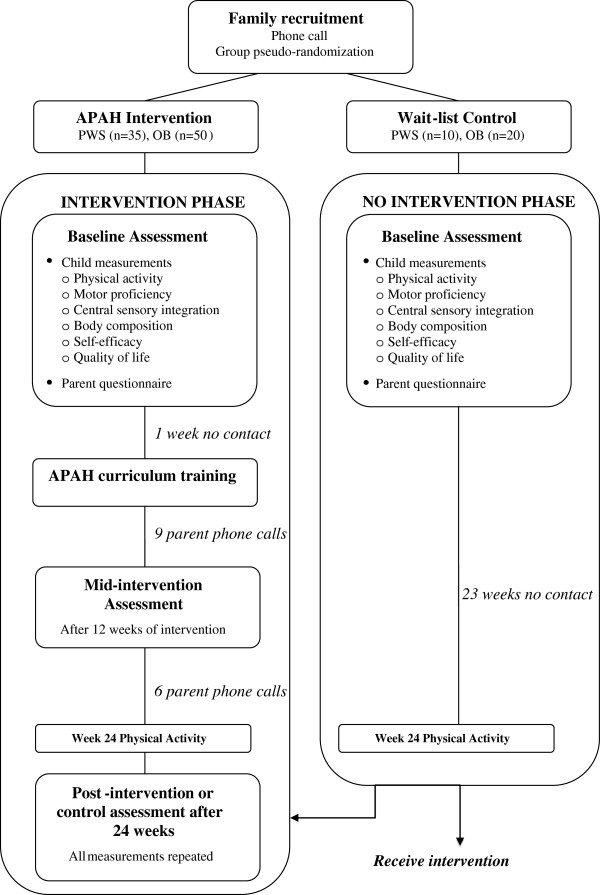
Study timeline for the Active Play @ Home (APAH) intervention.

The Active Play @ Home study will evaluate the effectiveness of a 24-week PA intervention on select motor and health–related parameters in children with PWS and children with obesity but without PWS. The study has a quasi-experimental design with semi-random assignment to an intervention group or a wait-listed control group. The intervention group will consist of families with a child with PWS (n=35) and families with a child who is obese (n=50). Ten additional families with a child with PWS will be assigned to a wait-list control group and 20 families with an obese child without PWS will also serve as a wait-list group. Both wait-listed groups will serve as control groups prior to being enrolled in the PA intervention. Groups of two to four families will complete all baseline and follow-up testing on the same day throughout the study. The semi-random assignment consists of a priori established participant cohorts for both intervention and control groups. Upon recruitment, participants will be provided with tentative dates for their visits and their randomization to the cohort (intervention vs. control) will be based on their date preferences. Ethic committee approvals have been obtained from the California State University Fullerton Institutional Review Board and the United States Army Human Research Protection Office. During the first visit, after all study procedures are verbally explained, participants will have time to read the informed assent and consent forms and ask questions regarding the procedures. Afterwards, children participants will sign the informed assent form and their parents or guardians will sign the informed consent form. Figure 1 presents a timeline of the study procedures.

### Intervention description

This home-based intervention was developed by a team of experts with backgrounds in physical education, physical activity and obesity prevention in children, psychological aspects of physical activity, and motor control/learning. The intervention will consist of providing parents and children with a physical activity curriculum and its accompanying equipment needed to engage in four days of PA per week for 24 weeks. In addition, parents will be trained on the delivery of the Active Play @ Home curriculum, and will receive follow up support phone calls throughout the intervention.

Formative work done prior to designing the intervention includes the completion of group interviews with 28 parents of children and adults with PWS (2-22 years old) and a questionnaire completed by 90 parents with a child with PWS [16]. The group interviews indicated that most parents understood the benefits and the therapeutic role of PA for their children. The most frequently raised issues were as follows: the physical limitations associated with PWS may make it difficult to engage in PA; the children may consider PA to be more work than fun and therefore not enjoy it; the children may get easily frustrated, especially when the activities are too difficult to perform successfully; the parents may find the time spent in PA with their children too burdensome; and the cost of a PA program may be too expensive. Parents’ preferences for PA mode, frequency, and location for a successful PA program included: 1) Activities that can occur at home, in a caring, supportive, and non-competitive environment that foster participation from all family members; 2) interventions that include a variety of fun activities such as interactive console games, goal-oriented games, and dancing; 3) PA intervention materials such as a handbook or DVD with ideas for activities and hands-on instructions; and 4) a flexible schedule. The questionnaire findings emphasized the need to incorporate vigorous activities in the curriculum since children with PWS tend to spend more time engaging in moderate PA and much less time performing muscle and bone strengthening activities [16].

Additional formative work included designing the set of progressive games and activities to be included in the curriculum, evaluating the feasibility of the assessment tools selected to measure the desired intervention outcomes, and then piloting the selected activities with children with and without PWS to ensure that they were appropriate for different skill levels, of adequate intensity, and met different space requirements. As a result of this formative work it was determined the need to obtain test-retest reliability data in children with PWS for the measurements of motor proficiency, central sensory reception and integration, self-efficacy and quality of life. In addition, the parents outcomes were further refined. Table 1 summarizes the different formative work and the objectives from each activity.

**Table 1:** Formative work done prior to development of the physical activity intervention “Active play @ home”

**Timeline**	**Activity**	**Participants**	**Objectives**
Nov 08 - May 09	4 Group interviews	28 parents of children with PWS	1) To determine if PA was viewed as a therapeutic tool for PWS. 2) To identify needs, concerns, and barriers for PA in PWS and key factors of a successful PA intervention.
Nov 08 - May 09	Mail in survey	Parents of people with PWS in CA (n=90)	1) Describe current PA involvement in people with PWS by age group to identify needs and gaps in participation. 2) Describe barriers and facilitators to PA.
January - March 2010	Preliminary curriculum design	Investigators (Wiersma, Rose, Rubin) and students (Junior and Schroeder)	1) Develop a skeleton of game-based activities to be included in the curriculum. 2) Select games from the Nintendo Wii Fitness that could be included in the curriculum.
April 2010	Visits to CSUF campus to pilot PA activities and assessments	Five children with PWS ages 10-16 y Four children without PWS ages 9-12 and their parents	1) Determine suitability, like, dislike, enjoyment, and level of exertion for select games to be included in curriculum, possible modifications for difficulty and space required. 2) Determine feasibility of assessment procedures to be used in the intervention in children with PWS.

### Curriculum and parental support

**Figure 2:**
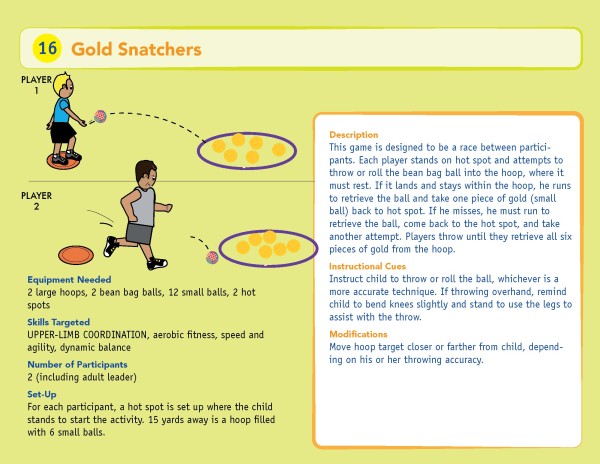
Sample playground game from the Active Play @ Home curriculum (Wiersma LD, Rubin DA, Rose DJ, Schroeder L, and M Junior, 2011).

The Active Play @ Home curriculum includes age-appropriate goal-oriented physical activities that combine playground and video games with specific exercises that target the following parameters: muscular strength and endurance, aerobic endurance, flexibility, balance, agility, and motor coordination. This curriculum was designed for children ages 8-11 years without disability and children ages 8-15 years with PWS. The curriculum includes playground games and interactive console-based games using the Nintendo Wii™, each of which are to be performed twice weekly. The duration of activity is systematically progressed from 25 to 45 minutes of MVPA over the course of the 24-week intervention. Both the parents and children will be trained to use the curriculum during a hands-on session at baseline. Each family receives the Active Play @ Home manual that contains a daily schedule detailing the activities for each of the four days of the week for the 24 weeks. The manual includes four sections: 1) introduction to the program that includes the philosophy guiding the program, a description of the motor skill and fitness components targeted, and a description of the parent’s role in leading the activities; 2) the daily schedule; 3) detailed descriptions of the playground games; and 4) instructions for playing the interactive console-based games. Each illustrated playground game is accompanied by instructions on how to carry out the activity, as well as instructional cues and modifications to assist the parent in leading the activities for his/her child (See Figure 2). In the interactive console-based games section of the manual we have included instructions on how to set-up the Nintendo Wii™ system and step-by step instructions for playing each of the selected games. Families receive all media equipment necessary as well as a variety of play balls, cones, hoops, foam mats, and boundary markers to be used throughout the intervention.

Over the 24-week program, parents will receive phone calls from a member of the research team who provides PA counseling and troubleshooting for parents regarding the implementation of the curriculum. Phone calls will take place every week during the first four weeks and then once every two weeks for the remainder of the intervention. In addition, the parents and children will complete physical activity checklists associated with each day of prescribed activity. The inclusion of self-monitoring techniques, along with discussions about planning and overcoming barriers into the parent training component of the program were considered to be important from the social cognitive theory perspective [24]. These regulatory skills of monitoring, planning/scheduling, and goal setting are incorporated into the baseline training of parents and during the follow-up phone calls [25]. The self-efficacy of children and parents will be targeted with the age-appropriate progressive nature of the curriculum that is designed to provide the opportunity for successful mastery experiences for the children. Further, self-efficacy in the children will be targeted through parents serving as skilled or learning models for the activity and providing verbal encouragement throughout the program.

### Measurements

Outcome measurements in children.

#### *Physical activity*

Physical activity will be measured using accelerometers, which provide detailed information on the temporal patterns (duration, frequency, and intensity) of PA. Data will be stored as acceleration counts recorded in 5-second epochs using the 4 MB GT3X (Actigraph, Pensacola, FL) triaxial activity monitor. This accelerometer model has excellent intra- and inter-instrument reliability across a wide range of accelerations and has been validated for use with children ages 10 to 15 years [53]. The accelerometer will be worn at the right hip and secured with an elastic belt. Children will wear the accelerometer for eight consecutive days (Sunday to Sunday) while awake. They will be instructed to remove the accelerometer for showering/bathing, swimming, or when going to bed. Initially the data will be screened for compliance of wear time (at least 10 hours per day on 3 weekdays and 1 weekend day). Non-wear periods will be defined as any period with 60+ consecutive minutes of missing data (0 counts). Because the families will participate in data collection on Saturdays, only accelerometry data collected on Sundays will be eligible for analyses for a weekend day. To determine thresholds for sedentary, light, moderate, and vigorous physical activity we will use the children cut-points established by Evenson et al. (2008) [54]. These cut-points were recently recommended as the best choice for categorizing physical activity intensities in children [55]. In addition, the parent with his/her child will also complete a log of all the physical activities the child engages in over the eight-day period.

#### *Motor proficiency*

Motor proficiency will be evaluated using the long form version 2 of the Bruininks-Oseretsky Test of Motor Proficiency (BOT-2) [56]. The BOT-2 is composed of 53 items and provides a total motor composite score across 4 domains: fine manual control (fine motor precision and fine motor integration), manual coordination (manual dexterity and upper limb coordination), body coordination (bilateral coordination and balance), and strength and agility (running speed and agility and strength). We expect to see baseline differences between the children and youth with PWS and those without PWS in all domains and composite areas. In addition, we expect significant improvements following completion of the PA intervention in the areas of upper limb coordination, bilateral coordination, balance, running speed, and agility and strength. As fine manual control and manual dexterity are not targeted in this intervention, change in these parameters will not be assessed. The BOT-2 is designed to identify individuals with mild-to-moderate coordination deficits and has been validated for use in 4–21 year olds [56]. A sub-sample of participants with PWS will complete the BOT-2 twice at baseline, separated by an interval of one week, to determine test-retest reliability.

#### *Sensory-motor reception and integration*

The evaluation of sensory reception and motor integration is an exploratory component of this study. The intent is to first establish the validity and reliability of the Sensory Organization Test® (SOT) when administered to children and adolescents with PWS on two days separated by a week in a small sub-sample of participants. The same sub-sample will also perform the SOT at regular interventions during the intervention period to determine if any changes in sensory reception and integration occur as a result of the intervention.

The SOT is designed to identify impairments in one or more of the three sensory systems (i.e., vision, somatosensory, vestibular) that contribute to standing balance. This test has been previously used with pediatric populations, with and without disabilities [57]. The test is comprised of six test conditions. *Condition one* serves as the control condition because there is no manipulation of the sensory environment. In *condition two*, vision is removed by having the participant wear a blindfold while standing on a stable support surface, whereas in *condition three*, vision is manipulated by sway-referencing the visual three-sided surround as the participant stands quietly on a stable support surface. In *condition four*, somatosensory inputs are manipulated by sway-referencing the support surface. Specifically, the support surface is rotated in an anterior or posterior (AP) direction in direct response to the sway generated by the standing participant. The ratio of support surface movement to AP sway of the participant is 1:1. In *condition five*, the support surface is once again sway-referenced but vision is also removed by having the participant wear a blindfold. Finally, in *condition six*, input from the visual and somatosensory systems are removed. For the SOT, all participants will be required to wear an overhead safety harness to ensure their safety during practice and test trials. Each participant will stand on the force platform of the Smart Balance Master® with the arms extended and to the sides of the body. The participants’ feet will be positioned in accordance with the manufacturer’s requirements for data collection (i.e., feet facing forward and the medial malleolus and the lateral calcaneus aligned with a grid superimposed on the surface of the force plate). The alignment of the feet is based on the individual’s height and done to ensure consistency of foot position across testing sessions.

During the first testing session, the test will be verbally explained to the participants after which they will physically perform one trial in each of the six sensory conditions. This practice trial is intended to familiarize them with the equipment, harness, and actual test protocol. Following a three-minute rest interval, participants will then perform three 20-second trials in each of the six sensory conditions. Participants will be permitted to rest as needed throughout the test.

#### *Anthropometric and body composition measures*

Anthropometric measures include body mass obtained to the nearest 0.1 Kg without shoes using an electric scale, and stature (measured without shoes, jackets, or other heavy clothing) at the end of inhalation to the nearest 0.1 cm using a wall-mounted stadiometer. BMI will be computed by dividing body mass (kg) by stature (m^2^) and BMI percentile values will be determined from the Centers for Disease Control growth charts [58]. Percentage of body fat and lean mass will be measured in a supine position using dual x-ray absorptiometry (DXA) scan (Lunar Prodigy Advance Plus; GE Healthcare, Milwaukee, WI). This specific DXA unit provides a reliable and accurate measurement of distribution of lean and fat mass [59]. We will perform regional fat mass and lean mass calculations, in addition to total body analysis, using the enCORE pediatric software version 12.30.008, which automatically demarcates the regional boundaries for all regions.

#### *Health-related quality of life*

The measurement of health-related quality of life provides information about physical, emotional, social, and school components of wellbeing based on the calculation of two summary scores (i.e., physical and psychological). For this measurement we will use the validated 23-item PedsQL™ 4.0 Generic Scale [60]. This measure has shown good reliability and validity in children and adolescents ages 2-18 years old [60]. In addition, parents will complete the parent version of the PedsQL scale. Both measurements will be used to evaluate changes in quality of life. For participants with PWS we will first determine the test-retest reliability of this instrument at two time intervals separated by one week to decide if this measurement is reliable and is acceptable for use.

#### *Child’s self-efficacy for physical activity*

Self-efficacy for physical activity will be measured with an eight item questionnaire rated on a five point scale ranging from disagree a lot to agree a lot [61-64]. This questionnaire was originally developed for use with children in fifth grade, but also validated with children in eighth grade [61-64]. This questionnaire had a test-retest reliability of r=0.84 over a period of two weeks. Additionally, the questionnaire had an internal consistency score of 0.88 [62,63]. For participants with PWS we will first determine the test-retest reliability of this instrument at two time intervals separated by one week to decide if this measurement is reliable and is acceptable for use.

#### *Dietary intake*

To help interpret whether changes in body composition can be solely attributed to the PA intervention, dietary intake will also be assessed at the same time points as the other variables of interest. The participating parent or legal guardian will maintain a food record of the child’s diet during two days of the week and one day on the weekend. In this record the parent will include quantity of food and fluids consumed, the preparation method, and the brand of the product. Before the baseline measurement, parents will attend a training session with a registered dietitian to learn how to estimate portion sizes and keep a food record. The information collected through the food records will be entered into The Food Processor, ESHA Research, Salem, OR, USA program and analyzed for macronutrient percent intake and total calories.

#### Parent measurements

The parent who consents to participate in the study will provide demographic (ethnicity, family income, occupation, primary language spoken at home, etc.) and medical information (e.g., child’s medical history, current conditions related to the child’s ability to engage in physical activity, medications use, intelligence scores in the case of children with PWS). Each parent will also complete several questionnaires to measure the following:

##### *Regulatory efficacy for the parent*

Parents’ confidence to regulate and manage their child’s PA will be assessed using a modified version of a proxy efficacy scale for regulating exercise behavior [65]. The original scale will be adapted to reflect the parents’ confidence to regulate and motivate their children to participate in PA. In a survey of parents of children aged 2-18 years, this modification of the proxy efficacy scale has shown excellent reliability (Cronbach Alpha=.98) and an acceptable one week test-retest reliability correlation of .64 [66].

##### *Parental influence*

This scale assesses the regulation (i.e., social control) used by parents to encourage PA behavior in their children [67]. This scale is separated into three types of social control. The two positive types of social control include positive (encouraging) and collaborative (being active with the child). The negative type of social control examines nagging and ordering to be active. In a recent study of parents of children ages 2 to 16 years the reliability of this measure has been shown to be acceptable-to-good for all types of social control: collaborative (Cronbach alpha=.77), positive (Cronbach alpha=.67) and negative (Cronbach alpha=.85) [68].

#### *Process measures*

Qualitative information will also be collected to further evaluate the feasibility of this type of PA intervention. Parents will be asked to provide information in the form of completed checklists addressing the implementation of the specific playground games in terms of difficulty and enjoyment by the children as well as total time spent per session doing the prescribed activities. This information will serve as a form of adherence monitoring as well as a means by which the families can provide feedback to the research team about the content of the intervention.

Additional information will be obtained during on-site feedback sessions conducted at 12- and 24-weeks of the intervention. Parents will be asked about barriers and facilitators for the implementation of the intervention, likelihood of participating in a future project, satisfaction with the study, the influence of participation in the study on their lifestyle, and their child’s overall participation in PA. Parents will also be asked to provide suggestions as to how the intervention could be modified to better serve their individual needs.

### Data analyses

The main purpose of this study is to investigate short-term changes (immediately following the 24-week intervention) in PA, motor proficiency, and health-related outcomes in children with and without disability following completion of a physical activity program conducted in the home environment. A 2 (time: pre, post) by 2 (group: control, intervention) by 2 (youth: obese or PWS) mixed model ANOVA will be used to assess changes in the primary outcome (i.e., physical activity). Similar analyses will also be performed to assess changes in gross motor proficiency, body composition, and central sensory reception and integration. Other changes that will also be evaluated using mixed model ANOVAs include changes in quality of life, child self-efficacy, parent regulatory efficacy, and parent social influences. Potential confounders will serve as covariates where appropriate.

A power analysis was performed using G*Power 3.1.9 to identify the appropriate sample size for a power of .95, an alpha level of .05 and a correlation between time points was assumed to be .50. A small to moderate effect size (*f*=.20) was chosen, given the variability in physical activity expected in children with PWS [15]. A sample size of 84 was identified to provide sufficient power to detect an interaction. Given a 30% probability of drop-outs, 115 children will be recruited.

## Discussion

The Active Play @ Home study differs from other home-based intervention studies because it provides parents and children with a well-rounded and systematically progressed game-based curriculum. Additionally, the 24-week PA intervention is much longer than previous studies (i.e., 3-weeks to 3-months). Moreover, in terms of intervention programs designed for children with disability, this study is unique in that it is delivered in the home environment with parents or caregivers serving as agents of change. Moreover, the games and exercises can be modified for larger or smaller play spaces and indoor or outdoor environments using minimal playground equipment.

Social influences received from family have frequently been identified as playing a key role in children’s physical activity behavior [29]. We have developed a curriculum that requires active involvement of the parents in providing PA opportunities for their children. In families that have more than one child, the implementation of this curriculum can also benefit other children in the family. Moreover, all activities can be adapted to be played with more children, in which case neighbors, cousins, or friends can participate.

Children who have a rare disease may have less opportunity to participate in appropriate PA in a group setting. Sometimes their motor skills or their intellectual ability present a hurdle for such participation. Individual PA programs may promote PA in an appropriate manner. Several strategies using console-based games have been tested in different populations with disability suggesting promising results. However, strategies that include a game-based curriculum including both playground games and console-based games to be played in the home environment such as the one we have designed have yet to be tested. This type of strategy needs to tested as it could provide an alternative option to common after-school programs to increase physical activity in children with and without disability.

## Abbreviations

PA: Physical activity; PWS: Prader-Willi syndrome; BMI: Body mass index; DXA: Dual x-ray absorptiometry; BOT: Bruininks-Oseretsky test of motor proficiency; SOT: Sensory organization test.

## Competing interests

The authors have no competing interests to declare.

## Authors’ contributions

DAR conceived the study, participated in its design and the development of the Active Play @ Home curriculum, and drafted the manuscript. KSW contributed to study procedures, selected the statistical analyses, and drafted sections of the manuscript. LW contributed to the study procedures, developed the playground games portion of the Active Play @ Home curriculum, provided edits for the manuscript. JWW contributed to study design and provided edits for the manuscript. DJR contributed to the design of the study as well as the development of the Active Play @ Home curriculum, provided edits for the manuscript. All authors read and approved the final manuscript.

## Authors’ information

DAR is trained in exercise physiology as well as in physical education. Her experience in physical activity intervention studies includes a multi-site middle school trial as well as two after school programs. DAR brings expertise on PWS and exercise. KSW is trained in exercise psychology with a background in promotion of physical activity and she contributes her knowledge of behavior change theories and social influence to this study along with her statistics background. LDW is trained in sport psychology with an emphasis in youth sports and has been involved with several grants promoting developmentally appropriate physical activity in children and adolescents. JWW is a clinical health psychologist with further training in preventive medicine. JWW contributes expertise in the area of family impact on children’s physical activity and overall wellbeing. DJR contributes expertise in the area of motor control and learning and has designed and implemented several intervention studies aimed at improving balance, gait, and overall motor performance.

## Pre-publication history

The pre-publication history for this paper can be accessed here:

http://www.biomedcentral.com/1471-2431/14/41/prepub
